# Targeting mutant p53 in cancer: the latest insights

**DOI:** 10.1186/s13046-019-1302-0

**Published:** 2019-07-05

**Authors:** Silvia Di Agostino, Giulia Fontemaggi, Sabrina Strano, Giovanni Blandino, Gabriella D’Orazi

**Affiliations:** 1 Oncogenomic and Epigenetic Unit, Department of Research, Diagnosis and Innovative Technologies, IRCCS Regina Elena National Cancer Institute, 00144 Rome, Italy; 2 Department of Medical Science, University ‘G. D’Annunzio, 66013 Chieti, Italy; 3 Unit of Cellular Networks and Molecular Therapeutic Targets, Department of Research, Advanced Diagnostic and Innovative Technologies, IRCCS Regina Elena National Cancer Institute, 00144 Rome, Italy

**Keywords:** p53, Li-Fraumeni, Small molecules, Mutant p53 reactivation, Exosomes, miRNA, Cancer-associated fibroblasts

## Abstract

This commentary wishes to highlight the latest discoveries in the mutant p53 field that have been discussed in the 8th p53 Mutant Workshop 2019, held in Lyon. *TP53* mutant (mutp53) proteins are involved in the pathogenesis of most human cancers. Mutp53 proteins not only lose wild-typ53 function but, in some circumstances, may acquire novel oncogenic functions, namely gain-of-function (GOF), which lead to aberrant cell proliferation, chemoresistance, disruption of tissue architecture, migration, invasion and metastasis. Decoding the *TP53* mutational spectrum and mutp53 interaction with additional transcription factors will therefore help to developing and testing novel and hopefully more efficient combinatorial therapeutic approaches.

## Background

**Fig. 1:**
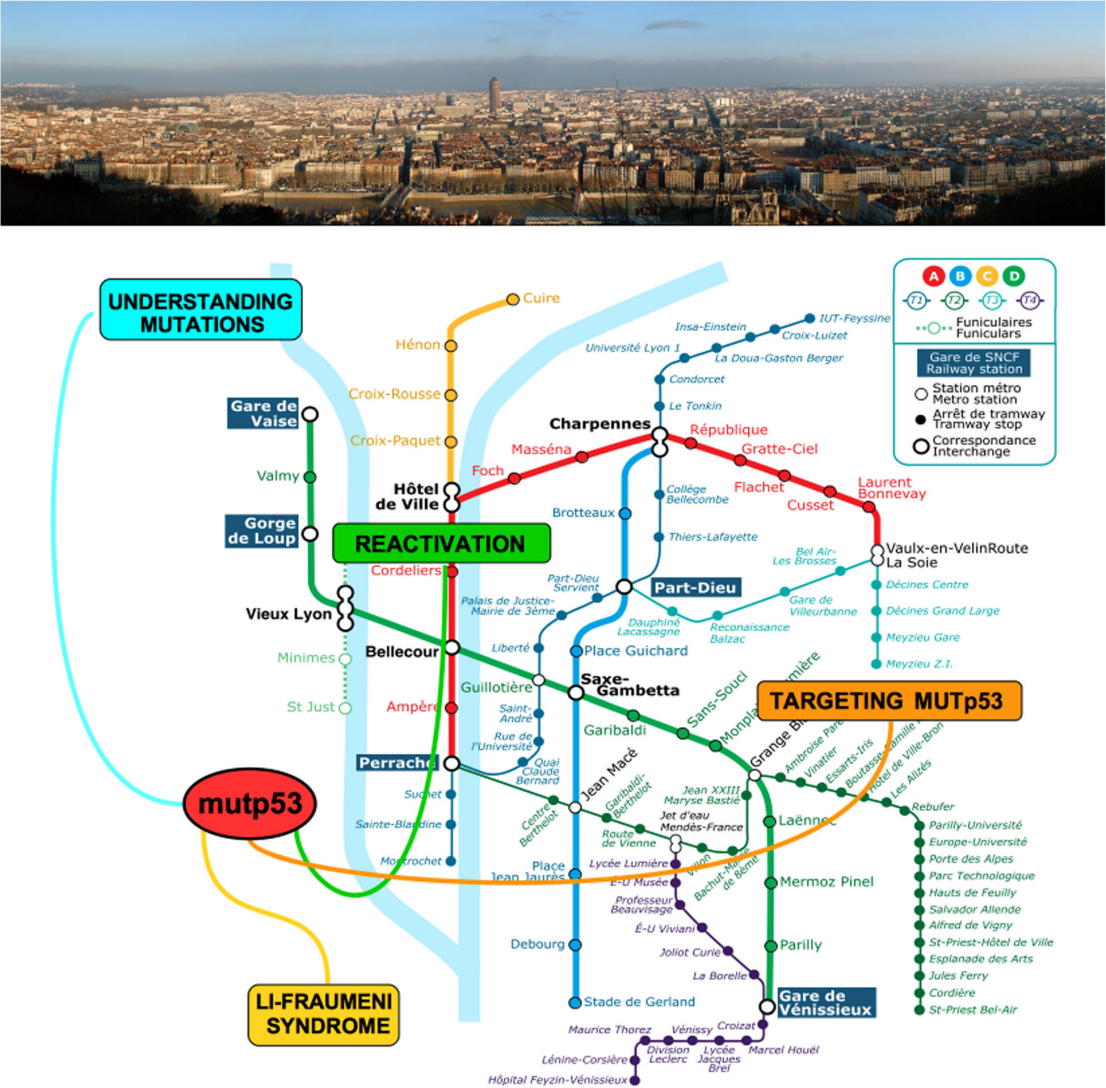
Schematic representation of the ongoing research in the mutp53 field. The Lyon’s map as a framework to show the crucial hub of mutp53 at the crossroads of many interventions aimed at understanding its mutational status, and the way to reactivate it for diagnostic and therapeutic purposes

*TP53* is the most frequently inactivated tumor suppressor gene in tumors, being mutated in over 50% of human cancer types and indirectly inactivated in many others. The loss of *TP53* as a signature driver of human cancers is unquestionable. Loss of p53 tumor suppressor functions induces accumulation of genomic alterations culminating in cancer progression, however, other than loosing wild-type (wt) oncosuppressor function, some mutant p53 (mutp53) proteins may acquire new oncogenic functions, namely gain-of-function (GOF), associated with altered p53-dependent transcriptional programs [1]. In addition, mutp53 interplay with other oncogenic transcription factors may profoundly alter the cancer genome and secretome dictating tumor progression even through remodelling of the tumor microenvironment [2]. The 8th p53 Mutant Workshop 2019 held in Lyon 15–18 May, has been the occasion to come across to the latest discoveries in the mutant p53 field. This occurred in a very special time for p53 studies since the Li-Fraumeni syndrome (LFS) was first described in 1969 and p53 itself came into being in 1979 while mutant p53 was recognized as a major event in cancer in 1989. Therefore, in this triple anniversary great progresses into understanding the multiple aspects of mutant p53 proteins can be underscored and, most importantly, how to exploit them in the next decades to target cancers with mutp53 accumulation (Fig. 1).

Important insight into the p53 field come from the Li-Fraumeni syndrome that was first described in 1969 as a highly penetrant cancer-prone syndrome inducing sarcoma, breast cancer, adrenocortical carcinoma, and brain tumors, often with more than one cancer per affected individual. More than 85% of patients with LFS harbour germline *TP53* mutations that profoundly affect the onset of the disease. In the years, the whole-exome and whole-genome testing allowed to sequence germline and somatic DNA leading to the beginning of precision medicine initiatives in LFS [3]. Although the clinical characteristics and molecular basis for LFS are now clear, no universally accepted approach exists for risk management. To this aim, recent studies showed that whole-body magnetic resonance imaging (WBMRI) may play a role in surveillance of this high-risk population and, more importantly, may help in localizing malignant tumors in childhood. Therefore, WBMRI may be a useful component of the routine baseline assessment of *TP53* mutation carriers in children and adults [4].

A new aspect in unveiling unpredicted mutp53 functions is its link with dysbiotic microbiota that is associated, for instance, with lung carcinogenesis, the number one cause of cancer deaths. Recent microbiome studies have demonstrated a contribution of bacteria to carcinogenesis in colon and lung, for instance. Starting from the hypothesis that somatic mutations together with cigarette smoke generate a chronic inflammatory microenvironment and that epidemiological evidence indicates an association between repeated antibiotic exposure and increased lung cancer risk, it has been shown that mutations in *TP53* are associated with the enrichment of a microbial consortia that are highly represented in squamous cell carcinoma (SCC) tumors, providing novel biomarkers for early detection [5]. The tumor-host interaction is therefore an important link in the progression of cancer and even in therapeutic failure. Intriguingly, it has been reported in colon cancer patients that mutp53 triggers reprogramming of macrophages by non-cell-autonomous mechanism toward M2 immunosuppressive phenotype, through miR-1246-enriched exosomes. Uptake of these exosomes by neighboring macrophages triggers their miR-1246-dependent reprogramming into a cancer-promoting state. These findings, associated with poor survival patients, strongly support a microenvironmental GOF role for mutp53 to promote cancer progression and metastasis [6]. The tumor microenvironment offers favourable conditions for tumor progression, and activated fibroblasts, known as cancer-associated fibroblasts (CAFs), play a pivotal role. Recently, a specific signature of microRNA (miRNAs) (miR-126, miR-141 and miR-21), which are called metastamir, was found upregulated in the serum with a significant correlation with the presence of early stage colorectal liver metastasis [7]. In light of the positive correlation between mutant p53 and miR-21 expression existing in different metastasizing tumors, is to be supposed that these findings might unveil additional microenvironmental GOF mechanisms for mutp53, although how mutp53 is involved in the exosome machinery needs further studies.

Genetic reconstitution of the function of p53 leads to the suppression of established tumours as shown in mouse models [8]. This strongly supports the notion that p53 reactivation by small molecules could provide an efficient strategy to rescue p53 mutants and reactivate their anti-tumor capacity through a variety of mechanisms [9]. Stabilization of mutp53 folding by Apr-246, which is currently being tested in a Phase II clinical trial, appears so far to be the most promising approach, when combined with various kinase inhibitors or inhibitors of PARP enzymatic activity. In the 8th p53 Mutant Workshop several classes of compounds that could reactivate wild-type p53 activities have been discussed, such as Mdm4 inhibitors, which are currently undergoing clinical testing, MdmX inhibitors and molecules targeting factors upstream of Mdm4/X [10]. Several new molecules and peptides, found through multiple approaches, have been proposed in the last years to restore a wild type conformation of mutp53, resuming previous studies indicating that ablation of the metallothioneins, which are zinc-storing proteins, leads to the unfolding of wtp53 and thus inhibits its transcriptional activity.

## Conclusion

At the end of the Workshop more questions than answers were raised, and it was clear that the successful implementation of p53-based therapies into clinical practice requires a thorough understanding of the mechanisms underlying the p53 response in both cancer cells and normal tissue (Fig. 1). Several important issues should be addressed in the future including the identification of biomarkers of resistance to p53-based therapies and possible side effects. The combinations of drugs that can act synergistically when combined with p53 reactivators seem a very promising approach.

## Data Availability

All data analysed in this study are included in this published article.

## Abbreviations

CAFs: Cancer-associated fibroblasts
GOF: Gain-of-function
LFS: Li-Fraumeni syndrome
miRNA: microRNA
mutp53: mutant p53
SCC: Squamous cell carcinoma
WBMRI: Whole-body magnetic resonance imaging
wtp53: Wild-type p53

## References

[1] Mantovani F, Collavin LDS (2019). G: mutant p53 as a guardian of the cancer cells. Cell Death Differ.

[2] D'Orazi G, Cirone M (2019). Mutant p53 and cellular stress pathways: a criminal alliance that promotes cancer progression. Cancers (Basel).

[3] Guha T, Malkin D (2017). Inherited TP53 mutations and the li-Fraumeni syndrome. Cold Spring Harb Perspect Med.

[4] Ballinger ML, Best A, Mail PL, Khincha PP, Loud JT, Peters JA (2017). Baseline surveillance in li-Fraumeni syndrome using whole-body magnetic resonance imaging: a meta-analysis. JAMA Oncol.

[5] Greathouse KL, White JR, Vargas AJ, Bliskovsky VV, Beck JA, von Muhlinen N (2018). Interaction between the microbiome and TP53 in human lung cancer. Genome Biol.

[6] Cooks T, Pateras IS, Jenkins LM, Patel KM, Robles AI, Morris J (2018). Mutant p53 cancers reprogram macrophages to tumor supporting macrophages via exosomal miR-1246. Nat Commun.

[7] Frixa T, Donzelli S, Blandino G (2015). Oncogenic MicroRNAs: key players in malignant transformation. Cancers (Basel)..

[8] Lozano G (2007). The oncogenic roles of p53 mutants in mouse models. Curr Opin Genet Dev.

[9] Blandino G, Di Agostino S (2018). New therapeutic strategies to treat cancers expressing mutant p53 proteins. J Exp Clin Cancer Res.

[10] Miranda PJ, Buckley D, Raghu D, Pang JB, Takano EA, Vijayakumaran R (2017). MDM4 is a rational target for treating breast cancers with mutant p53. J Pathol.

